# Whole-genome resequencing reveals new mutations in candidate genes for Beichuan-white goat prolificacya

**DOI:** 10.1080/10495398.2023.2258166

**Published:** 2023-09-20

**Authors:** Aimin Zhou, Yi Ding, Xiaohui Zhang, Yugang Zhou, Yadong Liu, Tingjian Li, Long Xiao

**Affiliations:** a Animal Husbandry Research Institute, Mianyang Academy of Agricultural Sciences, Mianyang, P. R. China; b Key Laboratory of Agricultural Animal Genetics, Breeding and Reproduction of Ministry of Education, Huazhong Agricultural University, Wuhan, P. R. China

**Keywords:** Whole-genome resequencing, goat, missense mutation, fecundity, candidate genes

## Abstract

In this study, we evaluated the copy number variation in the genomes of two groups of Beichuan-white goat populations with large differences in litter size by *F*_ST_ method, and identified 1739 genes and 485 missense mutations in the genes subject to positive selection. Through functional enrichment, *ITGAV, LRP4, CDH23, TPRN, RYR2* and *CELSR1* genes, involved in *embryonic morphogenesis*, were essential for litter size trait, which received intensive attention. In addition, some mutation sites of these genes have been proposed (*ITGAV*: c.38C > T; *TPRN*: c.133A > T, c.1192A > G, c.1250A > C; *CELSR1*: c.7640T > C), whose allele frequencies were significantly changed in the high fecundity goat group. Besides, we found that new mutations at these sites altered the hydrophilicity and 3D structure of the protein. Candidate genes related to litter size in this study and their missense mutation sites were identified. These candidate genes are helpful to understand the genetic mechanism of fecundity in Beichuan white goat, and have important significance for future goat breeding.

## Introduction

Capra hircus is widely used for its meat, milk and hides, and has very high economic value (1). Litter size (the number of kids in one birth) is closely related to the economics of goat breeding, but studies have shown the heritabilities of litter size related traits are low and is strongly influenced by epigenetic factors. Although through long-term natural selection and artificial intervention, some characteristic goats have been bred and the genetic resources of modern domestic goats have been expanded (2). However, it is still the focus of current research to search for new reproductive control candidate genes, carry out polymeric breeding with known reproductive genes to make breakthroughs in improving high fecundity goats, and meet the growing demand for animal products (3,4). Therefore, the ongoing search of related genes that affecting reproductive traits is essential in the field of livestock breeding (5,6).

Genome resequencing facilitates population genetic structure assessment, molecular conservation and improvement of local livestock breeds. It has become a reliable way to mine candidate genes for important traits in livestock. Thus, genome-wide resequencing data is used to identify genomic characteristics of domestication or artificial selection, and marker-assisted selection (MAS) based on major candidate genes to improve yield traits remains an important alternative to traditional breeding techniques. Recently, some progress has been made in the research on reproductive traits in goat and sheep, and high fecundity genes such as *CCNB1, GDF9, FecB, FecG* and *FecL* have been identified (7). Guan et al compared Dazu black goats with Nei Menggu cashmere goats and found that genes (*PAIP2B, CCDC64, EPB41L5, BIRC6*) found in Dazu black goats were correlated with reproductive traits (8). Liu et al analyzed whole-genome selection signals of Duolang sheep, small-tailed Han sheep and Mongolian sheep and found 143 genomic regions were subject to selection, and genes related to reproduction, horn type and ear development were screened out (9). Other studies identified were associated with milk production, fat deposition, meat quality and production by whole-genome resequencing (10). However, current genomic characterization of kidding traits in goats and resequencing gene screening are still insufficient.

Beichuan-white goat, a local goat breed that evolved in the specific ecological environment in China. It is distributed at the border of Tibet Plateau and Chengdu Plain. While adapting to the great temperature difference in different seasons, Beichuan white goat keeps the average kidding percentage of female goat above 200%, which shows its high breeding characteristics undoubtedly. Thus, it is ideal resource to screen the breeding genes. However, few studies on the genomic characteristics and genetic characteristics of Beichuan-white goat and litter size have not yet been mined. In addition, the protection and utilization of germplasm resources of this variety need to be paid more attention. Therefore, to elucidate the genetic basis of reproduction in Beichuan-white goats, this study selected 12 female goats from breeding base of Beichuan white goat producing area, distributed in two tails with an average litter size (in three consecutive birth records) and divided into two groups of high fecundity (n = 6) and low fecundity (n = 6) goats. Then, whole gene resequencing was performed to them. Candidate genes of litter size were screened by genome-wide selection signaling assays and these results were compared to identify genomic regions putatively under selection. This study is to provide a theoretical basis for the breeding of goat kidding traits through the mining of candidate genes or molecular markers for the number of kids in Beichuan-white goat, which is of great significance for the breeding of new varieties of goat with multiple kids.

## Materials and methods

### Animals and ethics

A total of twelves 2-3-year-old healthy female Beichuan-white goats, average body weight 37 ± 6 kg, were divided into high fecundity (n = 6) and low fecundity (n = 6) and placed in a house. The goat was ear tragged, fed three times per day, and given free access to water and salt.

The ethical approval for this study was obtained from the Ethical Committee of Mianyang Academy of Agricultural Sciences with permission number MAAS-2022-003. All efforts were made to minimize possible discomfort during blood collection.

### Goat genomic DNA extraction

Genomic DNA was extracted by standard phenol chloroform method. Briefly, a 2 mL blood sample was used for the next processing step. The erythrocyte lysate was added in equal proportions and mixed for 10 min, centrifuged for 10 min in 4 °C 6000 rpm, and the supernatant discarded. Then, 20ul proteinase K, 10% SDS 200 μL and STE 1 mL was added, well shaken and mixed, and digested in a water bath at 55 °C overnight. Subsequently, an equal volume of Tris saturated phenol was added and centrifuged; DNA extractant, centrifuged; 3 times the volume of anhydrous ethanol, centrifuged; 1 mL of 75% ethanol centrifuged. Finally, 200 μL of TE solution was appended to dissolve the DNA and −20 °C stored. DNA quality and concentration were determined by gel electrophoresis and UV spectrophotometer.

### DNA library construction and whole genomic resequencing

For genome resequencing, at least 1 μg of genomic DNA from each sample was used to construct a library with an insert size of 350 ∼ 500 bp. The fragmented DNA was modified, and PCR amplification was performed to construct a sequencing library. The quality-checked libraries were sequenced on Illumina’s HiSEquation 2000 sequencer with an average sequencing depth of 10x to obtain Sequenced Reads.

### Genome-wide analysis of genetic variation

#### Sequencing data filtering and sequence alignment

The raw reads are filtered by removing the reads with adapter, unknown sequences over 10, and low quality (Q ≤ 10). The sequencing quality was evaluated by FastQC software after filtration (11). The main assessment items included the number of reads, the overall quality and length distribution of each base in reads, and the content and preferred types of the A, T, G and C bases in reads. The 150-bp paired-end reads were mapped onto the goat reference genome (ARS1CGCA_001704415.1). Use the bwa index -a bwtsw command to build an index for the reference genome file. The samtools tool was used to convert the sam files into bam files in binary compressed format (12). Then, we used the samtools sort command to sort the compared files according to the reference sequence serial number. Finally, the reads of PCR duplicates in the file were marked by the MarkDuplicates command of the picard tool and created an index using CreateSequenceDictionary.jar.

#### Identification and annotation of genetic variation sites

Firstly, HaplotypeCaller command of gatk tool was used to extract genetic variation information (13). The SNPs were filtered by a series of thresholds with ‘VariantFiltration’ mode of GATK package-4.0.8.1. The condition on variant filtering is DP (Depth) < 10, QD (Quality score by depth) < 2.0, FS (Phred-scaled p-value using Fisher’s exact test) < 60.0, SQR (StrandOddsRatio) >4.0, MQ (Mapping quality score) <40.0, MQRankSum (Rank sum test for mapping quality of reference and alternative within reads) <−12.5, and ReadPosRankSum (Rank Sum Test for relative positioning of reference and alternative alleles within reads) <−8.0. Simultaneous identification of variant information for all samples used GenotypeGVCFs command (joint calling). Finally, the MergeVcfs command of GATK tool was used to combine the identification results of all chromosomes into a vcf file containing the variation information of all samples.

The vcf file was split into 30 files (1 ∼ 29, MT) by chromosome using the vcftools tool (14). To shorten the annotation time, all contents of the files need to be removed except chromosome number, base position, rs number and allele information. Annotation of genetic variation information was performed using the Ensembl Variant Effect Predictor (VEP) online tool of the Ensembl database (http://asia.ensembl.org/info/docs/tools/vep/index.html).

### Genome-wide selective signal detection

*F*_ST_ statistics infer the likelihood of selection by calculating the difference in allele frequency of a marker site between different populations. A larger value of the statistic indicates a greater likelihood of population segregation at the site and a greater polymorphism between populations; conversely, a smaller likelihood of population segregation. In this study, the vcftools tool was used to calculate the amount of *F*_ST_ between single-kid and multi-kid group goat populations, and the analysis strategy of sliding-window method was used to scan the whole genetic range (window-size: 100 kb, window-step: 10 kb). The Z - standardized transformation of *F*_ST_ value is performed by R program. The Z conversion formula of *F*_ST_ is as follows:

$$Z_{F_{\text{ST}}}=\frac{\left|F_{\text{ST}}-\mu F_{\text{ST}}\right|}{\sigma F_{\text{ST}}}$$

Where, $\mu F_{\text{ST}}$ is the population mean *F*_ST_, $\sigma F_{\text{ST}}$ is the standard deviation of *F*_ST_. Using R of qqman package mapped of Manhattan that Z(*F*_ST_) >2.326 areas were identified to the selected area (p < 0.01) (15).

### Gene ontology analysis

The annotation information of each locus in the selection fragment was extracted based on the whole genome annotation file. The corresponding gene symbol of each locus were uploaded to Ensembl Biomart database and the goat gene symbol was converted into the corresponding human gene symbol. Function of enrichment using Metascape online website (http://metascape.org/gp/index.html#/main/step1), and FDR p < 0.05 was used as a criterion to screen for enriched pathways, from which the reproduction-related items and the genes were selected.

### Screening for gene mutation sites

Vcftools was used to calculate the *F*_ST_ values of all the loci of the above selected genes. Manhattan was draw by the qqman program in R, and the loci with *F*_ST_ ≥0.27 were identified as the selected site. The non-synonymous mutation sites in the selected region were counted and the allele frequencies in low fecundity and high fecundity were calculated.

### Protein 3D structure analysis and physicochemical property prediction

The I-TASSER online program (https://zhanglab.ccmb.med.umich.edu/I-TASSER/) was used to predict the 3D structure of proteins (16). We use Ensembl database to get the sequence and location information of the mutant gene and convert the amino acids in the mutant location. Amino acid sequences of wild-type and mutant genes were then input into the I-TASSER online program to predict protein structure. Then, according to the α helix, β folding, irregular curling, and spatial conformation of the protein structure, we determined whether the protein structure have changed. Protein structures was visualized by PyMOL 2.4. ProtScale online tool (https://web.expasy.org/protscale/) predicted the hydrophilicity of proteins before and after mutation and the GraphPad Prism8.0 was used to draw map of hydrophobicity.

## Results

### Phenotype and population division

**Figure 1.**
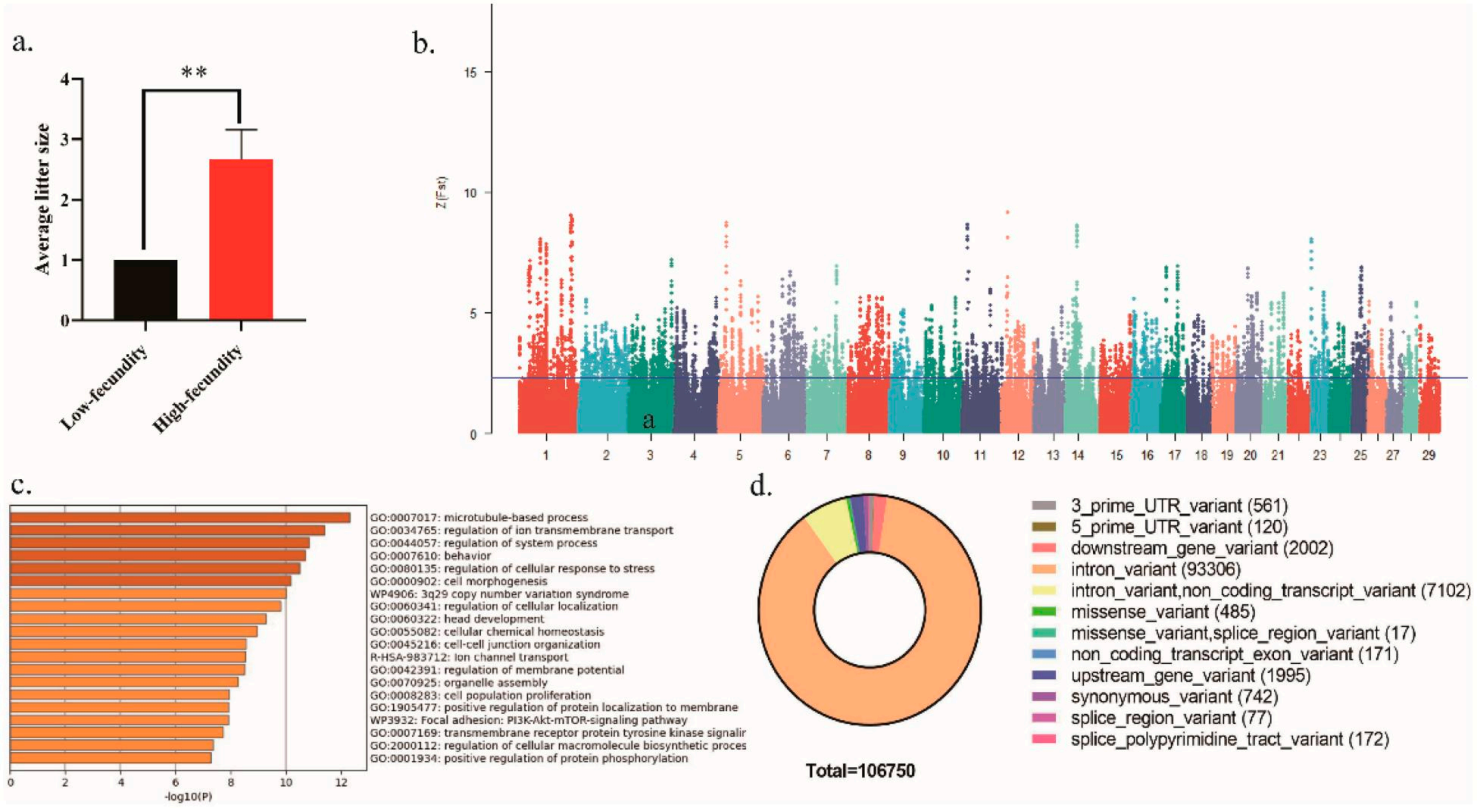
Genome-wide distributions of selection signals and reproduction-related candidate genes in two Beichuan-white goat populations. a) Distribution of average litter size of high and low fecundity Beichuan-white goat population. b) The positive end of the Z(*F*_ST_) distribution was plotted along Beichuan-white goat autosomes (different chromosomes are separated by color). the Z(*F*_ST_) values was calculated for each sliding 100Kb window with steps of 10Kb across the autosomes, and solid horizontal line indicates the cutoff (Z(*F*_ST_) >2.326) used for extracting outliers. c) The selected 1739 genes were enriched by the GO pathway using Metascape online database with the top 20 items shown, and q < 0.05 (p correction value) was the standard. d) Distribution of mutation types at mutation sites of the positively selected genes.

Figure 1 shows the descriptive statistics of litter size of Beichuan-white goat. For all selected goats with litter size records, ranged from 1 to 3, with a mean of 1.83 and a standard deviation of 0.91. As described above, 12 goats were divided into two population pairs according to the difference of phenotypic values. Of the 12 Beichuan-white goats, 6 of them were assigned to the high-fecundity (H) population with an average litter size of 2.67 ± 0.49, and the remaining 6 were assigned to the low-fecundity (L) population with an average litter size of 1.00 ± 0.00. As expected, the phenotypic values of litter size trait between H and L populations are significant differences (*P*-value <0.01) (Fig. 1a). The PCA indicated that the genetic backgrounds of H and L populations were similar and the population division based on the value of litter size did not lead to population stratification (Fig. 1).

### Whole genomic selective signal detection and enrichment analysis

To screen genomic regions under positive selection in high fecundity goats, a sliding window method based *F*_ST_ with a window size of 100Kb and a step size of 10Kb was calculated. The Manhattan plot was made through normalized *F*_ST_ results using Z(*F*_ST_)>2.326 as a threshold to filter selected regions (*p* < 0.01) (Fig. 1b). The results showed that 825 regions were positively selected region and the average length is 178.93Kb.

**Figure 2.**
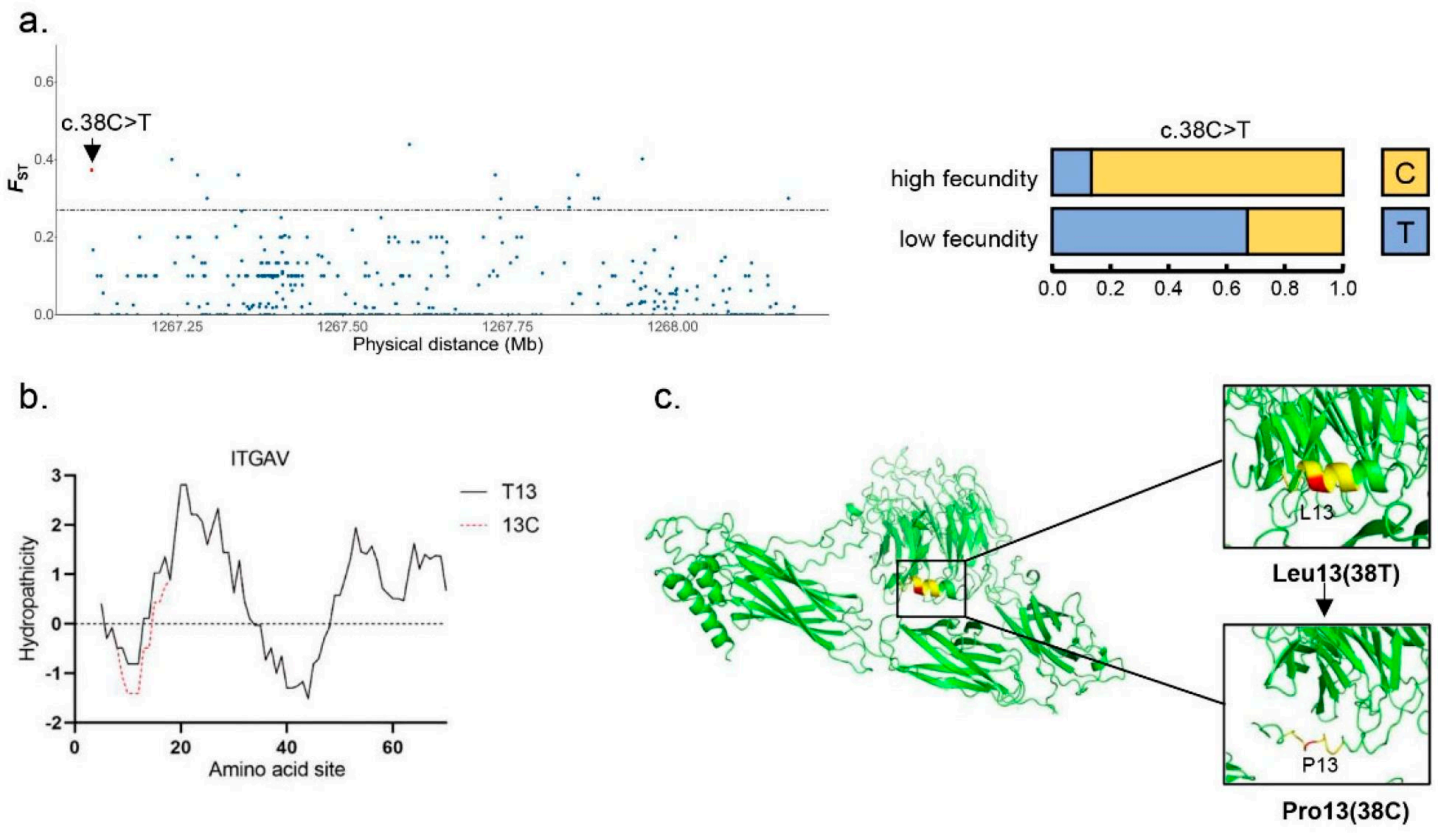
Result of selection signal detection and protein structure prediction on the *ITGAV* gene of Beichuan-white goats. a) Result of the *ITGAV* gene selection signal. The red dot represents the missense mutation site. The black solid line represents the selection signal screening threshold (*F*_ST_ =0.27). b) Effect of c.38C > T locus variation on the hydrophilicity of 0–20 amino acid sites in ITGAV. The black and red lines represent wild-type and mutant ITGAV proteins, respectively. c) The effect of c.38C > T locus mutation on the overall and local schematic 3D structure of wild-type and mutant ITGAV protein.

The positively selected regions screened correspond to 1739 genes, encoding protein (Table S1). Subsequently, these genes were subjected to functional enrichment analysis using Gene Ontology (GO) database. The top 20 of significantly enriched items are shown (Fig. 1c). GO pathway analyses identified the positively selected genes mainly enriched in *microtubule-based process*、*regulation of ion transmembrane transport, regulation of system process* and so on. In addition, genes in the top 5% of selected regions and their GO functional enrichment pathways were displayed and mainly enriched in *immunological synapse formation*, *regulation of vesicle-mediated transport*, *regulation of cell projection organization*, *cell-cell recognition* and so on (Table S2 and Fig. 2).

### Candidate genes related to litter size and SNP

There are diversified types of variation in positive selection sites, and the variation of these sites, especially non-synonymous mutations, may has a great impact on the 3-dimensional (3D) structure and physicochemical properties of proteins. Therefore, we further identified SNP of the positive selection genes screened in the above. The results showed that 485 missense mutation loci out of 106,750 mutation loci were identified in the chromosomes of these positive selection genes (Fig. 1d).

To understand the specific role of these missense mutations, functional enrichment of GO was performed. Importantly, some reproduction-related pathways such as *cell morphogenesis involved in differentiation*, *embryonic morphogenesis*, *and cell morphogenesis* were also significantly enriched (q < 0.05). Further, *ITGAV*, *LRP4*, *CDH23*, *TPRN*, *RYR2*, *CELSR1* genes related to these pathways were focused and identified as candidate genes affecting reproductive function in goats. In addition, some mutations interestingly appear to affect the auditory cells primarily.

### SNPs of genes associated with kidding under positive selection

According to the screened information on the autosomal mutation sites of these six genes, the recorded polymorphic sites were re-emphasized, and several new polymorphic sites were also proposed (Table 1).

**Table 1.** SNP Details of six reproductive related genes in autosomal sequence.

SNP	Database Number	Gene	Exon	Protein position	Amino acid variation	Alleles	Alleles frequency(single-kid)	Alleles frequency(multi-kid)
c.38C > T	novel	*ITGAV*	Exon 1	13	P/L	C/T	0.333/0.667	0.833/0.167
c.133A > T	novel	*TPRN*	Exon 2	45	T/S	A/T	0.000/1.000	0.833/1.167
c.1192A > G	novel	*TPRN*	Exon 4	398	R/G	A/G	0.200/0.800	0.750/0.250
c.1250A > C	novel	*TPRN*	Exon 4	417	Q/P	A/C	0.000/1.000	0.416/0.583
c.2042C > T	rs656514341	*TPRN*	Exon 7	681	P/L	C/T	0.300/0.700	0.750/0.250
c.5041C > T	rs661695950	*CDH23*	Exon 37	1681	V/I	C/T	0.583/0.417	1.000/0.000
c.7640T > C	novel	*CELSR1*	Exon 35	2547	V/A	T/C	0.083/0.917	0.500/0.500
c.5120T > C	rs638523001	*LRP4*	Exon 33	1707	V/A	T/C	0.500/0.500	0.917/0.083
c.2844T > G	rs652767212	*RYR2*	Exon 24	948	E/D	T/G	0.583/0.417	1.000/0.000

### Positive selection site mutation and protein structure prediction of ITGAV gene

One missense mutation of positive selection sites was detected in *ITGAV* gene, and the mutation localized in the first exon was shown in Fig. 2a. The ITGAV protein in the 13th amino acid occurred a mutation in the high-breeding group from Leu to Pro compared with low-breeding group, and the frequency of allele C of c.38C > T site in high fecundity goat population was 83.3%.

To further study the effect of selection signal on gene function, we predicted the influence of c.38C > T mutation of ITGAV on protein tertiary structure and hydrophilicity of amino acid residues. The results showed that the mutation caused a change of space structure from α- Helix to coil in the region of the mutation and their attachments (Fig. 2c). Meanwhile, it also led to the decrease of coefficient of hydrophilicity of amino acid residue from 0.111 to −0.489, resulting in the increase of hydrophilicity of nearby (Fig. 2b).

### Positive selection site mutation and protein structure prediction of TPRN gene

**Figure 3.**
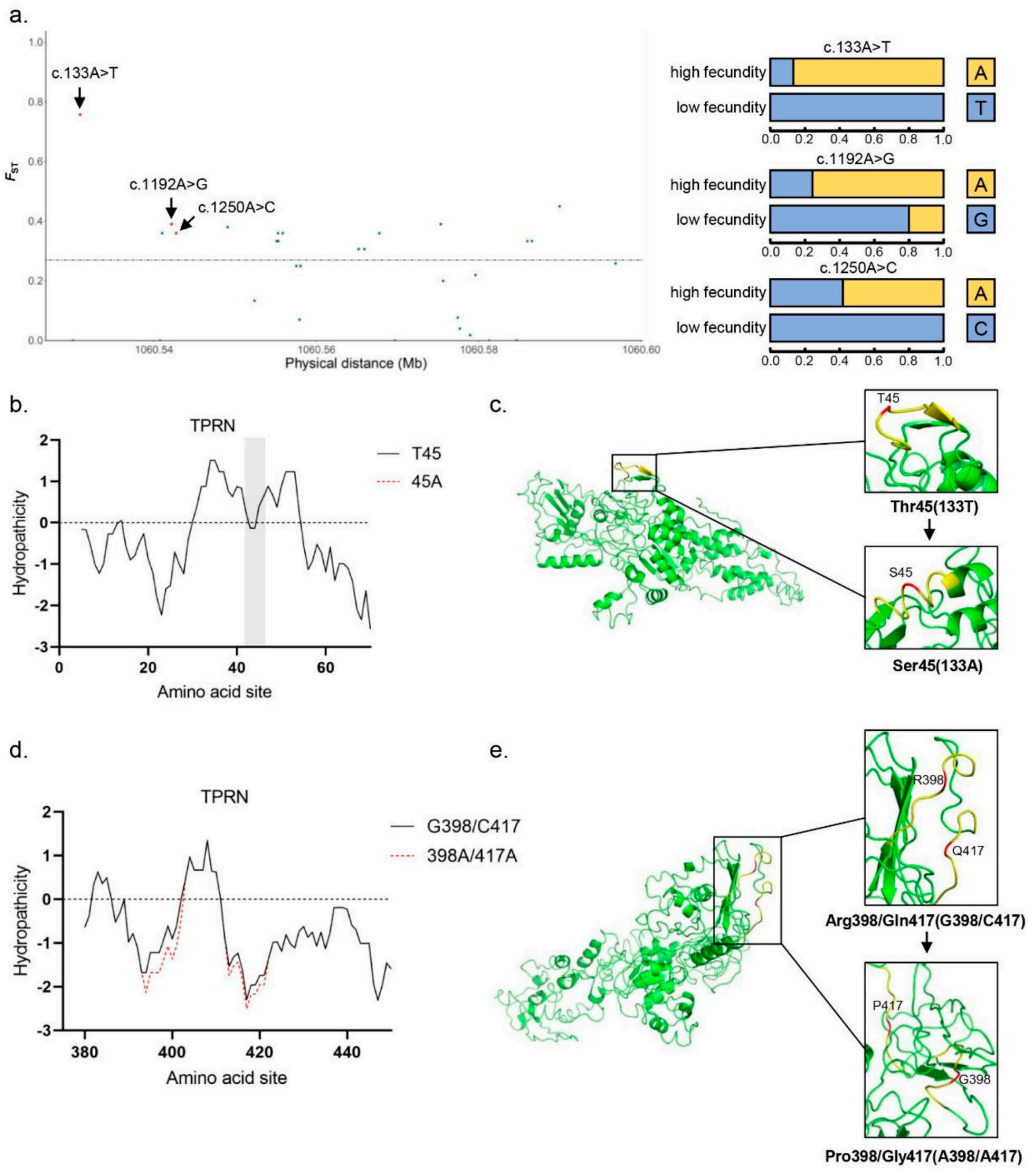
Result of selection signal detection and protein structure prediction on the *TPRN* gene of Beichuan-white goats. a) Result of the *TPRN* gene selection signal. The red dot represents the missense mutation site. The black solid line represents the selection signal screening threshold (*F*_ST_ =0.27). the effect of b) c.133A > T and d) c.1192A > G and c.1250A > C locus variation on the hydrophilicity of 40-60 and 380-440 amino acid sites in TPRN. The black and red lines represent wild-type and mutant TPRN proteins, respectively. The effect of c) c.133A > T and e) c.1192A > G and c.1250A > C locus mutation on the overall and local schematic 3D structure of wild-type and mutant TPRN protein.

By selective signal detection, three missense variants were found in positive selection sites in *TPRN* gene, including three unreported identified SNP sites (one located in the second exon and two located in the 4th exon) (Fig. 3a). Among the three mutations, allele A of c.133A > T, which corresponds to the 45th amino acid Thr, had a gene frequency of 83.8% in the high fecundity goats. However, allele T of c.133A > T corresponds to the 45th amino acid Ser in low fecundity goats had a gene frequency of 100%. In addition, in goats with high fecundity, the sites that c.1192A > G (p.398P > R) and c.1250A > C (p.417G > Q) were involved in arginine and glutamine, and the frequency of allele A increased to 75% and 41.6%, respectively.

The effect of mutations on the hydrophilic coefficient of amino acid site and 3D structure were analyzed. *TPRN* mutation showed that the variation of c.133A > T site only increased slightly the hydrophobicity of this site because its structure in this position inward spirals (Fig. 3b and c). The variation of c.1192A > G and c.1250A > C sites increased the hydrophilicity of the surrounding region significantly with the change of protein structure (Fig. 3d and e).

### Positive selection site mutation and hydrophilic coefficient prediction of CDH23, CELSR1, LRP4, RYR2 genes

**Figure 4.**
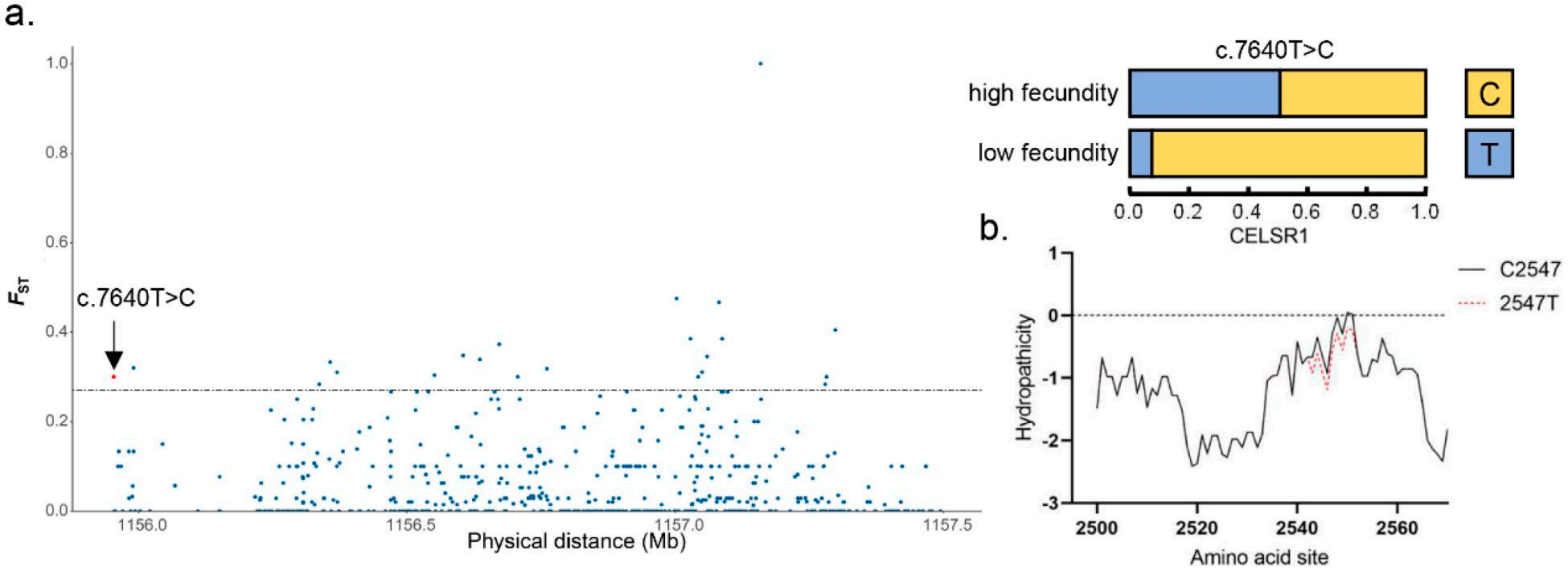
Result of selection signal detection and hydrophilicity coefficient on the *CELSR*1 gene of Beichuan-white goats. a. Result of the *CELSR1* gene selection signal. The red dot represents the missense mutation site. The black solid line represents the selection signal screening threshold (*F*_ST_ =0.27). b. Effect of c.7640T > C locus variation on the hydrophilicity of 2540–2560 amino acid sites in CELSR1. The black and red lines represent wild-type and mutant CELSR1 proteins, respectively.

More attention has been paid to the newly discovered genetic missense mutations. A mutation that called c.7640T > C has not been proposed in CELSR1 (Fig. 4a), which encodes two amino acids, Val and Ala. This mutation was found to cause a difference in allele frequency. As a consequence, the allele T frequency increased to 50% in high fecundity goats. The conversion from alanine to valine resulted in an increase in the hydrophilicity of the region around this site (Fig. 4b). Unfortunately, protein structure predictions of more than 1500 amino acids (AA) by the I-TASSER online program are generally considered inaccurate.

Missense mutations at positive selection sites (e.g.rs661695950, rs638523001 and rs652767212) have been put forward in some genes (*CDH23, LRP4, RYR2*) (Figs. 3a, 4a, and 5a). By predicting the change of hydrophilic coefficient, the results showed that among the reported genes, c.5041C > T caused a small increase of hydrophilicity in the region near this position of *CDH23* gene (Fig. 2b), while c.5120T > C had the opposite effect on *LRP4* gene in high fecundity goats (Fig. 3b). In addition, c.2844T > G had no effect on the hydrophilicity of *RYR2* gene (Fig. 4b).

## Discussion

### Genome-wide selection signal detection strategy

In the process of evolution, organisms are affected by selection, which manifests as changes in the frequency of alleles in individuals and populations, and manifests as changes in genetic diversity in gene structure. Long-term artificial selection has left genetic footprints in the goat genome adapt to different breeding goals (17). Some pathways that occurred in other species have been found to be frequent targets of selection. Therefore, it is theoretically feasible to identify character-specific selection traits by constructing strategies for population pairs with phenotypic differences. The advent of whole-genome sequencing and increasingly complete surveys of genetic variation help to investigate the regulation of genes under positive selection (18,19). The *F*_ST_ (Fixation Index) statistical approach, a selective sweep method, focuses on the amount of differentiation at a specific site of genome to determine whether selection is acting on the genomic region (20,21). Some genes related to some traits like coat color, growth, muscle development of goats have been identified with the boost of selected sweep analysis by whole-genome sequencing (22). Yao et al. conducted a whole-genome analysis on Huai goats and BoHuai goats, and identified a region on chromosome 7 that exhibited strong selection signals. Several genes related to important traits were detected in this region, including those associated with lipid metabolism (*LDLR*, *STAR*, *ANGPTL8*), reproductive ability (*STAR*), and disease resistance (*CD274*, *DHPS*, *PDCD1LG2*). These findings provide clues for further research on the important traits of Hua goats and BoHuai goats, and contribute to a better understanding of the genetic mechanisms underlying these traits (22). Chong et al. performed selection signal analysis by dividing Hu sheep into high-fertility and low-fertility groups, and identified 101 reproductive-related genes and 22 positively selected mutation sites (23). Tao et al. based on differences in reproductive ability, performed screening on YunShang black goat and discovered promising genes involved in ovarian function (*PPP2R5C*, *CDC25A*, *ESR1*, *RPS26*, and *SERPINBs*), seasonal reproduction (*DIO3*, *BTG1*, and *CRYM*), and metabolism (*OSBPL8*, *SLC39A5*, and *SERPINBs*) (24–27). Based on this, we applied *F*_ST_ method by whole-genome sequencing to assess genetic differentiation parameters between two populations of high and low reproductive Beichung-white goats. We found that several genes carrying selective traits were associated with litter size. Currently, the breeding of Beichuan-white goats mainly focuses on phenotypic selection, which involves selecting based on traits such as birth weight and number of offspring. However, this method has a slow breeding progress due to its susceptibility to environmental and nutritional factors, and it has limited genetic improvement potential. In contrast, the whole-genome selection approach used in this experiment provides a comprehensive examination of genetic variations associated with reproductive traits in goats. This approach enables a more detailed understanding of the underlying genetic mechanisms and accelerates the breeding progress of Beichuan-white goats.

Gene mutations could affect transcription, translation and mRNA transport, and missense mutations may alter the protein structure and lose its function, whereas sometimes the protein function is enhanced, thus improving the production traits of the animal (28–31). An et al. investigated polymorphisms of *GnRH1* and *GDF9* genes in different goat breeds, and a missense mutation was found in the *GDF9* gene had a significant effect on goat litter size by association analysis (32). In addition, two missense mutations (c.1457G > A and c.1645G > A) in *PRLR* gene have also been found to involve in regulating litter size in goats (33). Therefore, in this study, we focused on missense mutations in genes under the positive selection with a view to identifying proteins with potentially altered functions related to reproduction. This emphasis on functional genetic variation enhances our ability to identify specific genetic changes that may enhance reproductive traits in goats. However, while our study identifies candidate genes and missense mutations associated with reproductive traits, further experimental validation is required to confirm their functional impact on fertility. In addition, investigating gene-environment interactions could provide insights into how genetic variants related to reproduction respond to different environmental conditions, such as nutrition, temperature, and stress.

Among the GO-enriched pathways with missense mutations, *embryonic morphogenesis* was focused on. Changes in embryo morphogenesis are influenced by the embryonic genome, which is reprogrammed during the transformation of oocytes into embryos and before implantation (34). As a result, we identified *ITGAV, LRP4, CDH23, TPRN, RYR2 and CELSR1* genes related to this pathway as candidate genes. Some reports from different animals also confirmed that these genes were involved in reproduction related pathways (35,36).

### Genetic effect of c.38C > T mutation in ITGAV gene in goats

The human ITGAV protein, a member of the membrane bound receptor family, is involved in many developmental processes, including extracellular matrix mediated cell adhesion and migration, cytoskeletal organization, cell proliferation, survival and differentiation. It immobilizes cells to various substrates and strengthens external signals through membranes (37). During the early stages of pregnancy in livestock, the endometrium undergoes huge morphological and functional changes, and the embryo is regulated by adhesion so that it can adhere to the uterine wall, and because of this implantation, the placenta is formed. This period is accompanied by dynamic changes in gene expression (38,39). ITGAV is associated with cell adhesion and cell differentiation (40). ITGAV-containing heterodimers may be involved in TGFβ activation during embryo implantation through mechanisms of traction and Intracellular forces, and then active TGFβ could bind and activate TGFβRs at both embryo attachment and nonattachment sites (41). One study showed that placental ITGAV expression in late pregnancy was positively correlated with the number of live births in pigs (42). Therefore, in this study, the missense mutation of ITGAV was subject to positive selection, possibly due to the regulatory role played by this gene in reproduction.

Integrins such as ITGAV may exist in a high-affinity state when the fetus is attached to the maternal side. At this stage, biological forces such as tension, compression and shear forces play an important role in stabilizing the high affinity conformation of ITGAV, which are influenced by dynamic changes in maternal posture, movement and/or fetal position (43). ITGAV was found to increase in the luminal epithelium and trophectoderm of interplacentomal uterine and aggregate with several integrin-related proteins during pregnancy in sheep (44). The c.38C > T site mutation caused the change of the 13th amino acid of ITGAV, for proline was mainly found in high fecundity goats, whereas leucine in the low fecundity goats. Propensity of the change in this site in the high fecundity goats led to an increase in hydrophilicity near the amino acid residue (Fig. 2b), suggesting that ITGAV protein folding may be altered, thereby regulating its affinity state. Then, based on prediction, mutations in the ITGAV site were found to affect the 3D structure of the protein. The conformational change of ITGAV may affect its binding with proteases and affinity for fibronectin, and thus affecting cell adhesion. At the same time, cell migration and proliferation were affected by cell adhesion, which may affect embryonic stability (45).

### Genetic effect of missense mutation in TPRN gene in goats

The locus TPRN encodes a sensory epithelial protein, and mutations of which cause a progressive course of autosomal recessive nonsyndromic hearing loss (46). Wang et al. found that TPRN was involved in breast cancer prognosis, but the molecular mechanism remained to be elucidated (47). To date, few litter size related functions of TPRN have been reported and it has great potential as a biomarker for reproduction.

Our results showed four missense mutation sites for TPRN in high fecundity goats, one of which has been suggested. However, there was no direct evidence that this change was related to goat reproduction. Further research should be carried out to explore the protein conformation by amino acid transformations (Fig. 3c and e). For example, the 45th amino acid of TPRN was converted from threonine to serine by mutation at the c.133A > T site in high fecundity goats, and these two amino acids are often involved in the phosphorylation of proteins. Threonine and serine are usually attached by the phosphoric group and then activated by kinases, thereby regulating protein function and localization (48). In addition, some conserved amino acid residues (phenylalanine, tyrosine, arginine, etc.) on the protein surface have been identified to interact with DNA, suggesting that the c.1192A > G mutation (R/G) of TPRN in high fecundity goats may be involved in influencing intramolecular and intermolecular communication (49,50).

### Genetic effect of c.7640T > C mutation in CELSR1 gene in goats

The directional movements of the cilia enable the ovum to be transported steadily to the ampulla along with the fluid in the oviduct (51). They are regulated both within individual cells and between cells, but the mechanisms of regulation differ between animal species and organs (52). In mammalian ependyma, cilia direction is regulated by planar cell polarity (PCP) factors. CELSR1, a PCP factor and associated with sexual development, is asymmetrically located on the boundaries of each cell and is responsible for coordinating cilia orientation between cells (53). In addition, it could coordinate cilia direction of tissue-wide by aligning microtubule networks between cells. The researchers found that cilia orientation was not coordinated in *Celsr1*^−/−^ mutant oviducts (54). In this study, CELSR1 was identified as subject to positive selection in high fecundity goats, possibly due to its effect on cilia movements. Another reason contributing to the positive selection of CELSR1 is the importance of CELSR1 for the formation of fold pattern in the oviduct epithelium, which work together with cilia to improve transport efficiency. It was reported that the defect of CELSR1 resulted in ectopically-branched folds in mice and even infertility in humans and mice (55,56).

CELSR proteins consist of a large ectodomain (about 2700 amino acids), seven transmembrane segments and a cytoplasmic tail that is poorly conserved among the protein family. The ectodomain contains a G-protein-coupled receptor (GPCR) proteolytic site (GPS) embedded in the GAIN (GPCR Autoproteolysis INducing) domain (57). The GPS motif is an important element in receptor function (51). The crystal structure indicated that the GPS motif needed to be embedded in the GAIN domain to mediate autoproteolysis instead of acting by itself (57). The c.7640T > C mutation of CELSR1 in high fecundity goats is located in GAIN domain, and the increased hydrophilicity near this site may influence proteolysis and thus regulate function.

### Reported missense mutations in other genes

Although the missense mutations of *CDH23, LRP4* and *RYR2* genes identified in this study have been reported, there was no denying that some hints for our research were obtained by some interesting phenomena. For example, alleles of mutant sites in *CDH23* and *RYR2* genes (c.5041C > T and c.2844T > G) were completely fixed in highly fecundity goats, which infers a different direction of evolution. On the other hand, the amino acid corresponding to these sites of these genes was altered, whereas the hydrophilicity around these sites changed little, suggesting that they may not be critical to protein function.

## Conclusion

In this study, whole genome resequencing and selective signatures were conducted to identify candidate genes associated with kidding ability in phenotypically distinct population pairs of Beichuan white goats (high-fecundity and low-fecundity). The results indicate that 1739 positively selected genes were identified using FST analysis. GO pathway analysis revealed that these positively selected genes were primarily enriched in *microtubule-based processes*, *regulation of ion transmembrane transport*, *regulation of system processes*, and other functions. A total of 106,750 point mutations were found in the positively selected genes, including 485 missense mutations. The pathways involving missense mutations were predominantly associated with *differentiation*, *embryonic morphogenesis*, and *cell morphogenesis*. Importantly, the high fertility of Beichuan-white goats may be linked to specific positively selected genes (*ITGAV*, *LRP4*, *CDH23*, *TPRN*, *RYR2*, *CELSR1*). Among these genes, certain mutations (*ITGAV*: c.38C > T; *TPRN*: c.133A > T, c.1192A > G, c.1250A > C; *CELSR1*: c.7640T > C; *RYR2*: c.2844T > G; *CELSR1*: c.7640 > C) increased the hydrophilicity of the mutation sites, while *LRP4* (C.5120T > C) decreased hydrophilicity. This study deepened our understanding of the genomic characteristics underlying the kidding traits of Beichuan white goats and provided valuable guidance for further marker-assisted selection breeding and genomic selection breeding in goats. However, future studies incorporating genome-wide data from a larger number of individuals will be necessary to comprehend the complex interplay of factors involved in reproductive evolution.

## Supplementary Material

Supplemental Material

Supplemental Material

Supplemental Material

Supplemental Material

Supplemental Material

Supplemental Material

Supplemental Material
