# Supplementary material for: Whole-genome resequencing reveals new mutations in candidate genes for Beichuan-white goat prolificacya
Source: Anim Biotechnol. 2023 Sep 20;35(1):2258166. doi: 10.1080/10495398.2023.2258166 (PMC12674176; doi:10.1080/10495398.2023.2258166)
Supplement: Supplemental Material [file LABT_A_2258166_SM8382.docx]

Table S2 Genes in the top 5% of selected regions

| Item | Gene name |
| --- | --- |
| Genes in the top 5% of selected regions | *TSPO2*; *ADAM32*; *CHIC2*; *Y-RNA*; *SCARNA11*; *5S_rRNA*; *U6*; *FTMT*; *U4*; *Metazoa-SRP*; *OR6C74*; *SCARNA10*; *ZNF622*; *RREB1;* *NEK11;* *SNORA70*; *ADAM21*; *WDR53*; *DLG1*; *UNC5CL*; *MRPL51*; *MYO9A*; *ZNF786*; *CSMD3*; *VAMP1*; *SPAG17*; *TC2N*; *ATP2C1*; *NCAPD2*; *SH3GL3*; *COX16*; *TAPBPL*; *SMCO1*; *MED13L*; *FBLN5*; *FAM3B*; *GSG1L*; *HABP4*; *CCL19*; *NOP2*; *IFFO1*; *USP46*; *BACE2*; *ABCA13*; *PDIA4*; *RASEF*; *CHD4*; *KLHL29*; *ASTE1*; *RETREG1*; *XPO6*; *ALCAM*; *MELTF*; *AKAP6*; *OARD1*; *CCL21*; *APOBEC2*; *FBXO45*; *RYR2*; *OLFML2B* |
