# Supplementary material for: Whole-genome resequencing reveals new mutations in candidate genes for Beichuan-white goat prolificacya
Source: Anim Biotechnol. 2023 Sep 20;35(1):2258166. doi: 10.1080/10495398.2023.2258166 (PMC12674176; doi:10.1080/10495398.2023.2258166)
Supplement: Supplemental Material [file LABT_A_2258166_SM8381.docx]

Table S1 GO functional enrichment of missense mutations of the positively selected genes

| GroupID | Category | Description | Symbols | Log(p-value) | Log(q-value) |
| --- | --- | --- | --- | --- | --- |
| 1_Summary | Reactome Gene Sets | Sensory processing of sound by outer hair cells of the cochlea | WHRN,CDH23,XIRP2,TPRN,OTOG,ITGAV,LRP4,CHL1,RILPL1,ATP1B2,RYR2,CELSR1,SVEP1,OR52E1,OR5M3 | -5.89448 | -3.98599 |
| 1_Member | Reactome Gene Sets | Sensory processing of sound by outer hair cells of the cochlea | WHRN,CDH23,XIRP2,TPRN,OTOG | -5.89448 | -4.28702 |
| 1_Member | Reactome Gene Sets | Sensory processing of sound | WHRN,CDH23,XIRP2,TPRN,OTOG | -5.15425 | -3.72289 |
| 1_Member | GO Biological Processes | auditory receptor cell stereocilium organization | WHRN,CDH23,TPRN | -4.65495 | -3.34853 |
| 1_Member | GO Biological Processes | auditory receptor cell morphogenesis | WHRN,CDH23,TPRN | -4.49365 | -3.28414 |
| 2_Summary | Reactome Gene Sets | Ion channel transport | ATP1B2,RYR2,BSND,ATP13A4,ANO6,ATP13A5,CALB2,CDH23,ITGAV,PKDREJ,TSPO2 | -4.31069 | -3.18036 |
| 2_Member | Reactome Gene Sets | Ion channel transport | ATP1B2,RYR2,BSND,ATP13A4,ANO6,ATP13A5 | -4.31069 | -3.2473 |
| 1_Member | GO Biological Processes | auditory receptor cell development | WHRN,CDH23,TPRN | -4.16262 | -3.15722 |
| 1_Member | Reactome Gene Sets | Sensory processing of sound by inner hair cells of the cochlea | WHRN,CDH23,XIRP2,TPRN | -3.99884 | -3.04459 |
| 1_Member | GO Biological Processes | inner ear receptor cell stereocilium organization | WHRN,CDH23,TPRN | -3.72523 | -2.81675 |
| 1_Member | GO Biological Processes | inner ear auditory receptor cell differentiation | WHRN,CDH23,TPRN | -3.68494 | -2.81785 |
| 3_Summary | KEGG Pathway | Starch and sucrose metabolism | GYS2,MGAM,MGAM2,ATP1B2 | -3.57141 | -2.7421 |
| 3_Member | KEGG Pathway | Starch and sucrose metabolism | GYS2,MGAM,MGAM2 | -3.57141 | -2.77686 |
| 4_Summary | GO Biological Processes | phenol-containing compound metabolic process | OCA2,TG,TYRP1,GCNT4 | -3.57105 | -2.80869 |
| 4_Member | GO Biological Processes | phenol-containing compound metabolic process | OCA2,TG,TYRP1,GCNT4 | -3.57105 | -2.83865 |
| 1_Member | GO Biological Processes | hair cell differentiation | WHRN,CDH23,TPRN | -3.34142 | -2.63706 |
| 2_Member | GO Biological Processes | cellular calcium ion homeostasis | CALB2,RYR2,CDH23,ATP13A4,ATP13A5 | -3.31777 | -2.63973 |
| 1_Member | GO Biological Processes | inner ear receptor cell development | WHRN,CDH23,TPRN | -3.31185 | -2.65864 |
| 3_Member | KEGG Pathway | Carbohydrate digestion and absorption | ATP1B2,MGAM,MGAM2 | -3.22724 | -2.59751 |
| 2_Member | GO Biological Processes | regulation of cytosolic calcium ion concentration | CALB2,RYR2,CDH23 | -3.09823 | -2.49078 |
| 2_Member | GO Biological Processes | calcium ion homeostasis | CALB2,RYR2,CDH23,ATP13A4,ATP13A5 | -3.08799 | -2.50172 |
| 2_Member | GO Biological Processes | cellular metal ion homeostasis | ATP1B2,CALB2,RYR2,CDH23,ATP13A4,ATP13A5 | -3.07256 | -2.5065 |
| 5_Summary | GO Biological Processes | lipid transport | MTTP,SCP2,GRAMD1C,ABCA13,ANO6,TSPO2 | -3.0584 | -2.51165 |
| 5_Member | GO Biological Processes | lipid transport | MTTP,SCP2,GRAMD1C,ABCA13,ANO6,TSPO2 | -3.0584 | -2.53013 |
| 1_Member | GO Biological Processes | inner ear receptor cell differentiation | WHRN,CDH23,TPRN | -3.00421 | -2.49366 |
| 2_Member | Reactome Gene Sets | Ion transport by P-type ATPases | ATP1B2,ATP13A4,ATP13A5 | -2.98182 | -2.48831 |
| 2_Member | GO Biological Processes | cellular divalent inorganic cation homeostasis | CALB2,RYR2,CDH23,ATP13A4,ATP13A5 | -2.97927 | -2.50215 |
| 1_Member | GO Biological Processes | mechanoreceptor differentiation | WHRN,CDH23,TPRN | -2.89628 | -2.43496 |
| 6_Summary | GO Biological Processes | calcium ion transport | ITGAV,RYR2,PKDREJ,CDH23,ANO6,ATP1B2,IFNGR2,DPP6,BSND | -2.83635 | -2.39026 |
| 6_Member | GO Biological Processes | calcium ion transport | ITGAV,RYR2,PKDREJ,CDH23,ANO6 | -2.83635 | -2.40499 |
| 5_Member | GO Biological Processes | lipid localization | MTTP,SCP2,GRAMD1C,ABCA13,ANO6,TSPO2 | -2.82653 | -2.40941 |
| 2_Member | GO Biological Processes | cation transmembrane transport | ATP1B2,ITGAV,RYR2,PKDREJ,ATP13A4,ANO6,TSPO2,ATP13A5 | -2.72505 | -2.32172 |
| 2_Member | GO Biological Processes | divalent inorganic cation homeostasis | CALB2,RYR2,CDH23,ATP13A4,ATP13A5 | -2.71974 | -2.32977 |
| 1_Member | GO Biological Processes | sensory perception of sound | WHRN,CDH23,TPRN,OTOG | -2.64037 | -2.26336 |
| 1_Member | GO Biological Processes | cell morphogenesis involved in differentiation | ITGAV,LRP4,CHL1,WHRN,CDH23,TPRN,RILPL1 | -2.62894 | -2.26453 |
| 5_Member | GO Biological Processes | sterol transport | MTTP,GRAMD1C,TSPO2 | -2.58976 | -2.23758 |
| 2_Member | GO Biological Processes | cellular cation homeostasis | ATP1B2,CALB2,RYR2,CDH23,ATP13A4,ATP13A5 | -2.58769 | -2.24741 |
| 6_Member | GO Biological Processes | positive regulation of cation transmembrane transport | ATP1B2,IFNGR2,RYR2,ANO6 | -2.58189 | -2.25319 |
| 2_Member | GO Biological Processes | metal ion homeostasis | ATP1B2,CALB2,RYR2,CDH23,ATP13A4,ATP13A5 | -2.56065 | -2.24323 |
| 1_Member | GO Biological Processes | retina homeostasis | ATP1B2,WHRN,CDH23 | -2.55853 | -2.25211 |
| 7_Summary | GO Biological Processes | plasma membrane bounded cell projection assembly | TCTN2,ARMC9,ANO6,SPAG17,RILPL1,FSIP2 | -2.54462 | -2.24891 |
| 7_Member | GO Biological Processes | plasma membrane bounded cell projection assembly | TCTN2,ARMC9,ANO6,SPAG17,RILPL1,FSIP2 | -2.54462 | -2.25938 |
| 2_Member | GO Biological Processes | cellular ion homeostasis | ATP1B2,CALB2,RYR2,CDH23,ATP13A4,ATP13A5 | -2.50778 | -2.23276 |
| 8_Summary | GO Biological Processes | cell-cell junction organization | PATJ,WHRN,SVEP1,XIRP2 | -2.49856 | -2.23353 |
| 8_Member | GO Biological Processes | cell-cell junction organization | PATJ,WHRN,SVEP1,XIRP2 | -2.49856 | -2.24329 |
| 7_Member | GO Biological Processes | cell projection assembly | TCTN2,ARMC9,ANO6,SPAG17,RILPL1,FSIP2 | -2.46154 | -2.21582 |
| 6_Member | GO Biological Processes | positive regulation of ion transmembrane transport | ATP1B2,IFNGR2,RYR2,ANO6 | -2.45437 | -2.21798 |
| 1_Member | GO Biological Processes | sensory perception of mechanical stimulus | WHRN,CDH23,TPRN,OTOG | -2.4457 | -2.21845 |
| 9_Summary | GO Biological Processes | locomotory behavior | CHL1,VPS13A,CDH23,OTOG | -2.39481 | -2.17652 |
| 9_Member | GO Biological Processes | locomotory behavior | CHL1,VPS13A,CDH23,OTOG | -2.39481 | -2.1853 |
| 1_Member | GO Biological Processes | embryonic morphogenesis | ITGAV,LRP4,RYR2,CELSR1, CDH23,TPRN | -2.39386 | -2.19294 |
| 1_Member | GO Biological Processes | epidermis development | LRP4,WHRN,CDH23,SVEP1,TPRN | -2.37684 | -2.18436 |
| 6_Member | GO Biological Processes | calcium ion transmembrane transport | ITGAV,RYR2,PKDREJ,ANO6 | -2.35383 | -2.16962 |
| 6_Member | GO Biological Processes | regulation of potassium ion transmembrane transport | ATP1B2,DPP6,ANO6 | -2.32307 | -2.14698 |
| 7_Member | GO Biological Processes | cilium assembly | TCTN2,ARMC9,SPAG17,RILPL1,FSIP2 | -2.31766 | -2.14953 |
| 5_Member | GO Biological Processes | phospholipid transport | MTTP,SCP2,ANO6 | -2.31065 | -2.15036 |
| 1_Member | GO Biological Processes | inner ear morphogenesis | WHRN,CDH23,TPRN | -2.26234 | -2.10973 |
| 1_Member | Reactome Gene Sets | Sensory Perception | WHRN,CDH23,OR52E1,XIRP2,OR5M3,TPRN,OTOG | -2.2262 | -2.08115 |
| 2_Member | GO Biological Processes | cellular homeostasis | ATP1B2,CALB2,RYR2,CDH23,ATP13A4,ANO6,ATP13A5 | -2.21878 | -2.08115 |
| 6_Member | Reactome Gene Sets | Stimuli-sensing channels | RYR2,BSND,ANO6 | -2.18263 | -2.05229 |
| 10_Summary | WikiPathways | Complement system in neuronal development and plasticity | C1R,ITGAV,PATJ | -2.18263 | -2.05947 |
| 10_Member | WikiPathways | Complement system in neuronal development and plasticity | C1R,ITGAV,PATJ | -2.18263 | -2.06653 |
| 11_Summary | Reactome Gene Sets | Neutrophil degranulation | ITGAV,MGAM,OLFM4,DPP7,ABCA13,ANO6 | -2.16511 | -2.05596 |
| 11_Member | Reactome Gene Sets | Neutrophil degranulation | ITGAV,MGAM,OLFM4,DPP7,ABCA13,ANO6 | -2.16511 | -2.0628 |
| 6_Member | GO Biological Processes | positive regulation of ion transmembrane transporter activity | ATP1B2,IFNGR2,RYR2 | -2.16089 | -2.06532 |
| 7_Member | GO Biological Processes | cilium organization | TCTN2,ARMC9,SPAG17,RILPL1,FSIP2 | -2.153 | -2.06405 |
| 2_Member | GO Biological Processes | cation homeostasis | ATP1B2,CALB2,RYR2,CDH23,ATP13A4,ATP13A5 | -2.15245 | -2.07004 |
| 6_Member | GO Biological Processes | regulation of potassium ion transport | ATP1B2,DPP6,ANO6 | -2.15018 | -2.0742 |
| 2_Member | GO Biological Processes | inorganic ion homeostasis | ATP1B2,CALB2,RYR2,CDH23,ATP13A4,ATP13A5 | -2.11508 | -2.04545 |
| 6_Member | GO Biological Processes | positive regulation of transmembrane transport | ATP1B2,IFNGR2,RYR2,ANO6 | -2.07818 | -2.01479 |
| 12_Summary | GO Biological Processes | detection of stimulus | RYR2,PKDREJ,WHRN,LXN,OR52E1,PGLYRP3,OR5M3 | -2.05977 | -2.00255 |
| 12_Member | GO Biological Processes | detection of stimulus | RYR2,PKDREJ,WHRN,LXN,OR52E1,PGLYRP3,OR5M3 | -2.05977 | -2.00862 |
| 1_Member | GO Biological Processes | ear morphogenesis | WHRN,CDH23,TPRN | -2.04862 | -2.00345 |
| 6_Member | GO Biological Processes | positive regulation of transporter activity | ATP1B2,IFNGR2,RYR2 | -2.04862 | -2.00936 |
| 2_Member | GO Biological Processes | ion homeostasis | ATP1B2,CALB2,RYR2,CDH23,ATP13A4,ATP13A5 | -2.04684 | -2.01342 |
| 1_Member | GO Biological Processes | cell morphogenesis | ITGAV,LRP4,CHL1,WHRN,CDH23,TPRN,RILPL1 | -2.04634 | -2.01867 |
| 13_Summary | GO Biological Processes | homeostasis of number of cells | GCNT4,EMCN,LRRC19,TSPO2 | -2.01451 | -1.99252 |
| 13_Member | GO Biological Processes | homeostasis of number of cells | GCNT4,EMCN,LRRC19,TSPO2 | -2.01451 | -1.99812 |
| 2_Member | GO Biological Processes | cellular chemical homeostasis | ATP1B2,CALB2,RYR2,CDH23,ATP13A4,ATP13A5 | -2.00806 | -1.99721 |
| 14_Summary | GO Biological Processes | inflammatory response | IFNGR2,CIITA,RARRES2,SIGLEC1,LXN,ANO6 | -2.00043 | -1.99503 |
| 14_Member | GO Biological Processes | inflammatory response | IFNGR2,CIITA,RARRES2,SIGLEC1,LXN,ANO6 | -2.00043 | -2.00043 |
