# Supplementary material for: Whole-genome resequencing reveals new mutations in candidate genes for Beichuan-white goat prolificacya
Source: Anim Biotechnol. 2023 Sep 20;35(1):2258166. doi: 10.1080/10495398.2023.2258166 (PMC12674176; doi:10.1080/10495398.2023.2258166)
Supplement: Supplemental Material [file LABT_A_2258166_SM8370.docx]

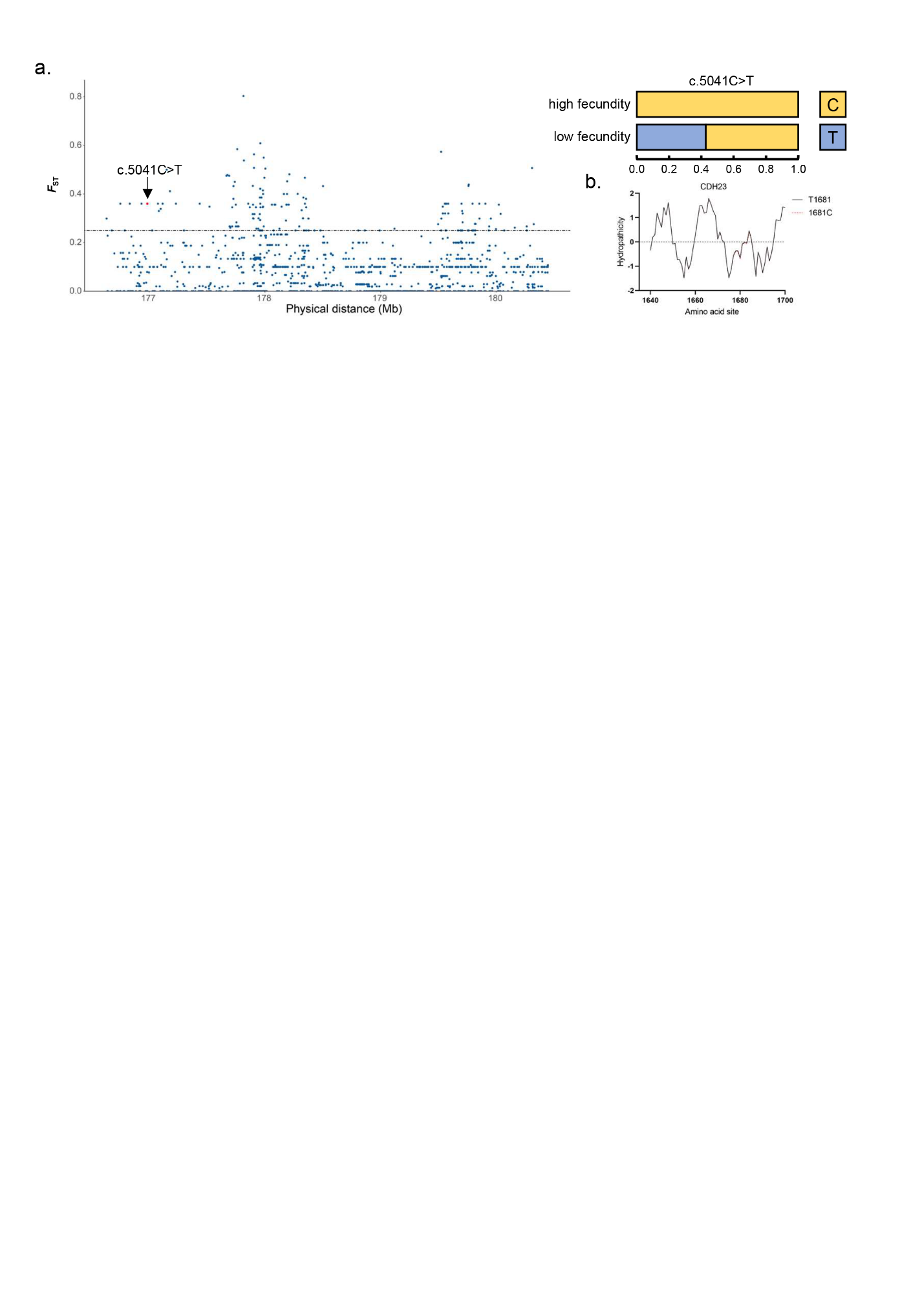
Fig. S3. Result of selection signal detection and hydrophilicity coefficient on the *CDH23* gene of Beichuan white goats. a) Result of the *CDH23* gene selection signal. The red dot represents the missense mutation site. The black solid line represents the selection signal screening threshold (*F*_ST_ =0.27). b) Effect of c.5041C>T locus variation on the hydrophilicity of 1660–1700 amino acid sites in CDH23. The black and red lines represent wild-type and mutant CDH23 proteins, respectively.
