# Supplementary material for: Whole-genome resequencing reveals new mutations in candidate genes for Beichuan-white goat prolificacya
Source: Anim Biotechnol. 2023 Sep 20;35(1):2258166. doi: 10.1080/10495398.2023.2258166 (PMC12674176; doi:10.1080/10495398.2023.2258166)
Supplement: Supplemental Material [file LABT_A_2258166_SM8369.docx]

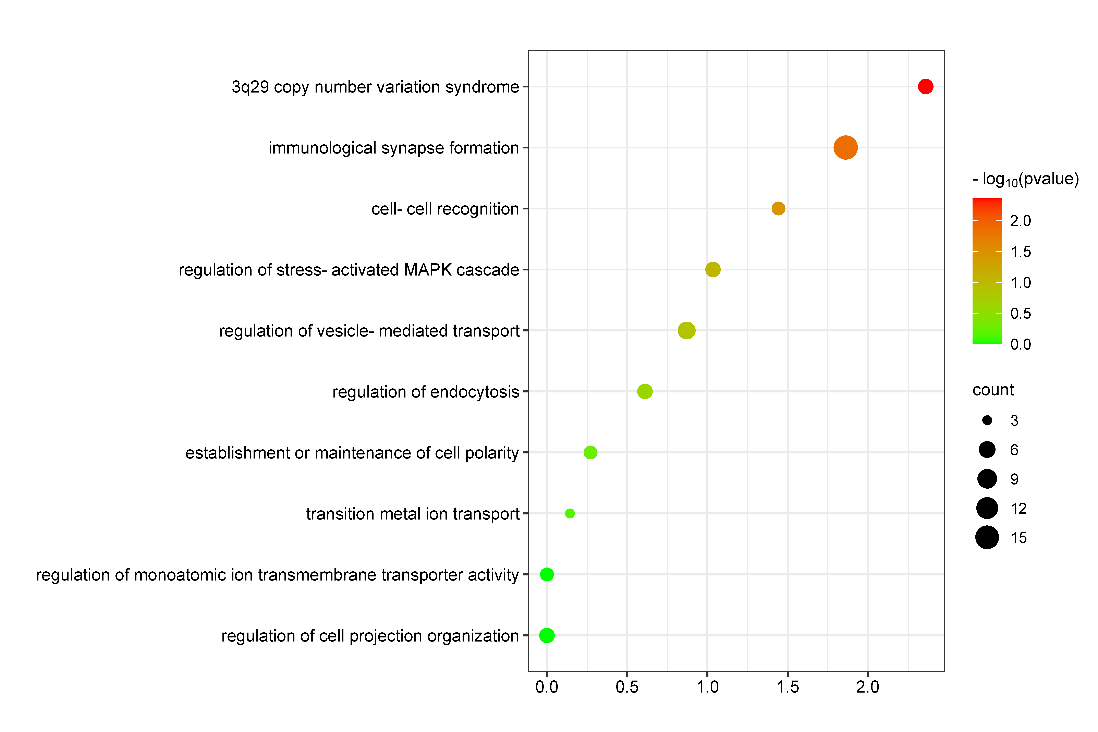
Figure S2. Genes in the top 5% of selected regions were enriched by the GO pathway using Metascape online database with the top 10 items shown.
