# Supplementary material for: Whole-genome resequencing reveals new mutations in candidate genes for Beichuan-white goat prolificacya
Source: Anim Biotechnol. 2023 Sep 20;35(1):2258166. doi: 10.1080/10495398.2023.2258166 (PMC12674176; doi:10.1080/10495398.2023.2258166)
Supplement: Supplemental Material [file LABT_A_2258166_SM8367.docx]

**
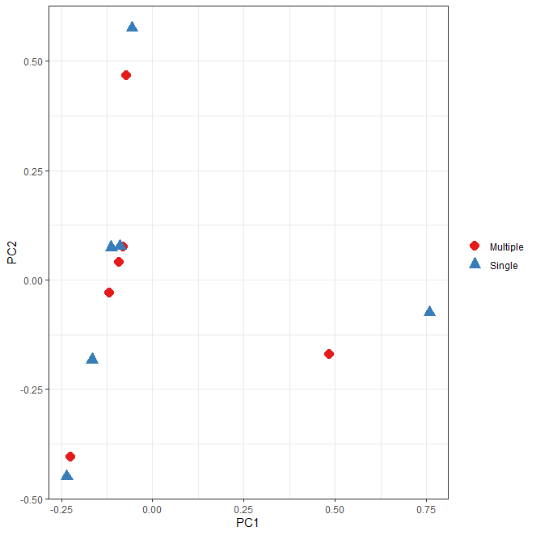
**

Figure S1. Principal analysis of multiple kid and single kid goats. The boxes mark the population of multiple kid, and the triangles mark the population of single kid.
